# Stressful Effects of Individual and Combined Exposure to Low-Concentration Polylactic Acid Microplastics and Chromium on Marine Medaka Larvae (*Oryzias melastigma*)

**DOI:** 10.3390/toxics12080594

**Published:** 2024-08-16

**Authors:** Yuan Yin, Yini Ma, Qiang Li, Nan Chen, Shaobai Wen

**Affiliations:** 1 NHC Key Laboratory of Tropical Disease Control, School of Tropical Medicine, Hainan Medical University, Haikou 571199, China; 18283467677@163.com (Y.Y.);; 2 School of Environmental Science and Engineering, Hainan University, Haikou 570228, China; 3 Hainan Ecological Environmental Monitoring Center, Haikou 570100, China

**Keywords:** marine medaka larvae, polylactic acid, chromium, ecotoxicology

## Abstract

Microplastics and heavy metal pollution frequently co-occur in the marine environment, raising concerns about their potentially harmful impacts on marine fish. This study undertook a comprehensive evaluation of the individual and combined stress effects of polylactide microplastics (PLA-MPs) and chromium (Cr) on marine medaka larvae. Following a 14-day exposure to PLA-MPs (100 μg/L) and Cr (50 μg/L), both individually and in combination, significant increases in heart rate and body length were observed. Notably, the combined exposure to PLA-MPs and Cr caused marked histopathological alterations, including shedding, atrophy, and lysis of the intestinal tissues. Furthermore, both individual and combined exposure induced oxidative stress in fish larvae, leading to changes in various enzyme activity indices. Individual exposure to either PLA-MPs or Cr led to anxious behavior in the larvae, whereas combined exposure not only caused anxious behavior but also altered swimming patterns. These findings suggest that combined exposure to PLA-MPs and Cr can exacerbate the toxic effects on marine medaka larvae.

## 1. Introduction

Microplastic (MP) pollution is a pervasive issue in the marine environment [1,2], with far-reaching ecological risks that have received great attention. The ingestion of MPs by aquatic organisms, such as fish and shellfish, has been shown to result in bioaccumulation and biomagnification, thereby posing potential risks to human health [3,4]. MPs have been reported to exist in a diverse range of organisms, from zooplankton [5] to fish [6] and marine benthos [7]. The ingestion of MPs can lead to a range of adverse effects, including oxidative stress and neurotoxicity [8], reproductive and metabolic toxicity [9], intestinal damage and alterations to the microbial community [10], as well as changes in locomotive behavior [11].

Polylactic acid (PLA), a biodegradable polymer, possesses desirable mechanical properties that set it apart from traditional plastic alternatives. The production of PLA has experienced steady growth and is expected to exceed 300,000 tons by 2024 [12]. However, studies have shown that PLA is difficult to degrade in natural environments at room temperature [13]. Therefore, PLA is subject to mechanical weathering, microbial degradation, and assimilation [14], and interacts with biological communities [15] to form MPs, which have a negative impact on aquatic organisms. Research has shown that both petroleum-based and bio-based MPs can inhibit the growth of microalgae, among which PLA-MPs exhibit the highest inhibitory rate [16]. Furthermore, PLA-MPs have been found to exert greater adverse effects on the gut of zebrafish than polyethylene terephthalate (PET), resulting in specific changes in the structure and diversity of the gut microbiota [10]. Nevertheless, the exposure concentrations adopted in these studies are high and do not accurately reflect the effects in real-world environments.

Heavy metals pose a significant threat to aquatic ecosystems due to their non-biodegradable nature and persistence in the environment. The toxic effects of heavy metals on fish are primarily mediated through the production of reaction oxygen species (ROS) via oxidation, producing an excess of oxygen [17]. Studies have shown that Cr^6+^ induces oxidative stress and immune responses, resulting in toxic effects on various aquatic organisms, including zebrafish [18], grass carp [19], and clams [20]. Exposure to lead has been shown to cause significant bioaccumulation in specific fish tissues, leading to oxidative stress, neurotoxicity, and alternations in immune function [21]. Arsenic, a common heavy metal pollutant in aquatic environments, has also been found to induce ROS production and inhibit acetylcholinesterase (AChE) activity, resulting in neurotoxic effects [22]. A recent study by Fred-Ahmadu found 26 heavy metals, including highly toxic substances such as chromium (Cr), copper (Cu), cadmium (Cd), lead (Pb), arsenic (As), and titanium (Ti), in MPs from coastal sediments in the Gulf of Guinea, with Cr concentrations ranging from 0.06 to 0.1 mg/kg [23].

It is essential to note that the interaction between MPs and heavy metals can significantly affect the bioaccumulation and toxicity of heavy metals [24]. Studies have shown that the presence of MPs can enhance the toxicity of Cd to zebrafish, with combined exposure leading to oxidative damage and inflammation of zebrafish tissues [25]. Conversely, another study examining the combined toxic effects of MPs and Cr on *Daphnia magna* revealed that MPs increased the toxicity of Cr in acute toxicity tests, while MPs tended to reduce the toxicity induced by Cr in chronic toxicity tests [26]. Therefore, the impact of MPs on metal toxicity is still controversial and needs further investigation. Recent compositional analyses of MPs isolated from the ocean have shown that MPs contain varying concentrations of metallic elements that may be harmful to the environment, marine life, and human health [27]. PLA-MPs not only have adverse effects on aquatic animals [10,28] but also possess the ability to bind to other pollutants, such as organic pollutants and heavy metals, serving as a carrier of more complex pollutants into organisms [29,30]. Co-exposure to PLA-MPs with copper and zinc has been shown to induce toxic effects on catfish and reduce fish immunity [30]. As the production of PLA plastics continues to increase as a substitute for petroleum plastics, it is necessary to conduct further studies to evaluate the biological toxicity effects of joint exposure to PLA-MPs and heavy metals. This is essential for expanding our understanding of the potential ecotoxic risks associated with PLA-MPs, which are currently understudied in the context of joint exposure.

The early stages of life are more sensitive to environmental pollutants, which can have far-reaching consequences for individual and population health [13]. In aquatic environments, Cr concentrations range from 3 μg/L to 40 mg/L, mainly in the form of trivalent chromium (Cr^3+^) and hexavalent chromium (Cr^6+^) [26]. Notably, Cr^6+^ exhibits higher toxicity due to its ability to permeate cell membranes. In this study, we utilized concentrations of 100 μg/L PLA and 50 μg/L Cr^6+^, both individually and in combination, to evaluate their stress effects on marine medaka larvae. The marine medaka larvae were chosen as a model organism due to their established utility in assessing the accumulation and toxicity of MPs [4,31,32]. The chosen concentrations are considered environmentally relevant [26,33]. Through experiments, we examined the impacts of PLA-MPs and Cr on the growth and development, oxidative stress (including SOD, CAT, and GSH enzyme activities), and locomotive behavior of medaka larvae. This study aims to expound on the negative effects of exposure to PLA-MPs and Cr, both individually and in combination, at concentrations reflective of ambient conditions, on the early life stages of organisms.

## 2. Materials and Methods

### 2.1. Experimental Materials

**Figure 1:**
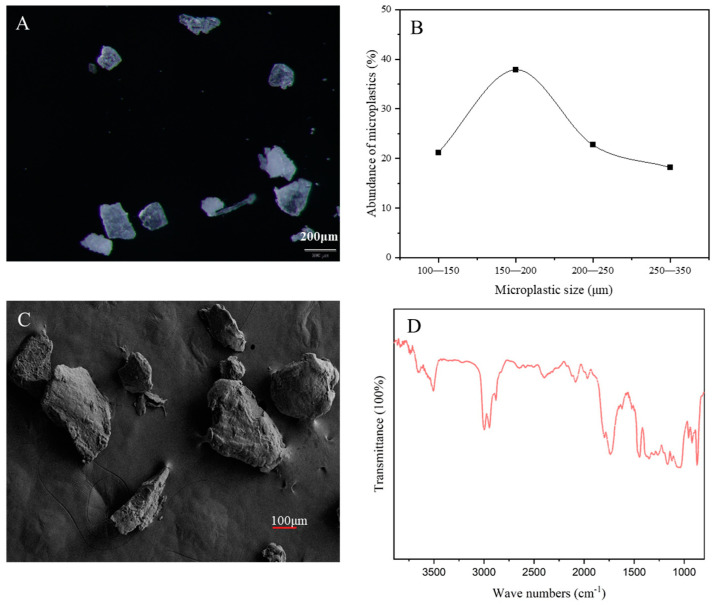
Characterization of MPs traits and composition. (**A**) Morphological analysis of PLA under stereomicroscopy; (**B**) particle size distribution of PLA-MPs; (**C**) scanning electron microscopy analysis of PLA morphology; (**D**) compositional analysis of PLA-MPs via micro-FTIR.

PLA-MPs were purchased from Dongguan Mingyuxing Plastic Raw Materials Co., Ltd. (Dongguan, China). and subjected to characterization utilizing a stereomicroscope (Olympus, SZX16+DP17, Tokyo, Japan), micro FTIR spectroscopy (ThermoFisher, Nicolet™ iN10, Waltham, MA, USA), and scanning electron microscope (ZEISS EIGMA, Oberkochen, Germany). The results of these analyses confirmed that the composition of the PLA-MPs was predominantly PLA, with particle sizes ranging between 100 and 300 μm. Notably, 3.03% of the particles were found to be smaller than 100 μm. Furthermore, morphological analysis revealed that the microplastics exhibited a fragmented structure (Figure 1). The marine medaka (*Oryzias melastigma*) used in this study were hatched and reared in-house within the laboratory and have been maintained in the laboratory for more than three generations. All reagents used in this experiment were of analytical grade, unless otherwise specified.

### 2.2. Preparation of Experimental Reagents

Preparation of Nile Red Stain: A quantity of 5 mg of Nile Red was accurately weighed out using an electronic balance (Mettler Toledo, XSR205DV, Greifensee, Switzerland) and dissolved in 10 mL of acetone solution to obtain a Nile Red Stain with a concentration of 500 μg/mL. Preparation of fluorescent PLA: A 0.1 g sample of PLA was placed in a 10 mL glass vial, and about 0.5 mL of the aforementioned Nile Red Stain solution was added to the mixture. The mixture was agitated and then placed in a fume hood until the acetone had completely evaporated and the mixture had dried naturally. Preparation of 1 mg/L Cr^6+^ standard solution: A quantity of 0.2829 g of solid potassium dichromate, previously dried in an oven at 105 °C for 2 h, was precisely weighted out using an electronic balance (Mettler Toledo, XSR205DV, Greifensee, Switzerland). The potassium dichromate was then dissolved in a fixed volume of 100 mL of water to obtain a potassium dichromate stock solution with a concentration of 1000 mg/L. Subsequently, 10 mL of the stock solution was diluted in a 1 L volumetric flask to obtain a 10 mg/L Cr^6+^ standard working solution.

### 2.3. Maintenance of Marine Medaka Larvae

Marine medaka larvae (15 days old) were cultured in artificial seawater (filtered to 0.45 μm) under controlled environmental conditions. The temperature was maintained at 26 ± 1 °C, with a photoperiod of 14 h of light and 10 h of darkness. The artificial seawater had a salinity of 35‰, the pH was 8.1 ± 0.1, and the dissolved oxygen content was 6.3 ± 0.1 mg/L. Larvae were fed twice a day, with lab-hatched brine shrimp provided in the morning and commercial feed in the afternoon. All animal experiments were conducted after obtaining ethical approval from the Animal Ethics Committee of Hainan Medical University(Grant No. HYLL-2024-694).

### 2.4. PLA Intake and Excretion Experiments

The intake and excretion experiments were performed in 2 L glass beakers (Figure S1). The experimental design consisted of three treatment groups: control (artificial seawater), fluorescent PLA-MPs (100 µg/L), and fluorescent PLA-MPs (100 µg/L) in combination with Cr^6+^ (50 μg/L). Prior to the addition of larvae, each exposure solution was sonicated for 30 min to ensure the uniform dispersion of MPs and Cr^6+^ in the artificial seawater. Five fish larvae were added to each exposure solution, and the solution was replaced every 48 h. Three replicates were set for each treatment group. On days 1, 3, 5, and 7, three fish larvae were randomly selected from each exposure group and examined under a fluorescence microscope (Olympus, IX83P2ZF, Tokyo, Japan) to assess MP ingestion. After 7 days of exposure, larvae from each treatment group were transferred to clean artificial seawater for excretion experiments. The artificial seawater was replaced every 24 h, and larvae were examined using fluorescence microscopy and photographed on days 1 and 2 of the efflux experiment to determine the rate of MP efflux. During the intake and excretion experiments, the feeding regimen for the larvae remained consistent with the protocol outlined in Section 2.3.

### 2.5. Exposure Experiment

Prior to the exposure test, fish larvae were subjected to a 2-day fasting period while being cultured in artificial seawater. Then, they were randomly allocated to twelve 2 L glass beakers, each containing 12 fish larvae and 1 L of exposure solution. The exposure experiment consisted of four treatment groups: control, 50 μg/L Cr^6+^ treatment, 100 µg/L PLA-MPs treatment, and a combined treatment of 50 μg/L Cr^6+^ and 100 µg/L PLA-MPs (Figure S1). Three replicates were set for each treatment, and the culture and feeding conditions were consistent with those described in Section 2.3. The exposure duration was 14 days. During the experiment, each culture tank was cleaned, and the exposure solution was replaced every 48 h. On the 7th and 14th days of culture, growing indices, physiological and biochemical indexes, and behavioral parameters were measured.

### 2.6. Growth and Development, Intestinal Sectioning, and Biochemical Analysis

The heartbeats of larvae were observed and recorded under a microscope for 1 min, with three replicates per fish and three fish per treatment. Body length was measured using vernier calipers (MEGUQMNT, Shanghai, China), with eight fish measured per treatment. In addition, whole-mount HE staining was performed on larvae due to their small size, with three fish larvae processed per treatment. The fish larvae were fixed in a paraformaldehyde solution (Wuhan Xavier Biotechnology Co., Ltd., Wuhan, China), dehydrated, paraffin-embedded, sectioned, and stained. All slides are viewed under a microscope (Nikon, Nikon Eclipse E100, Tokyo, Japan) and imaged using an imaging system (Nikon, NIKON DS-U3, Tokyo, Japan) and 3DHISTECH’s Slide Converter software for photography and image processing (the details of steps are presented in the Supporting Information). Biochemical assays were conducted to measure the levels of glutathione (GSH), catalase (CAT), and superoxide dismutase (SOD) in fish larvae using commercial kits (Jiancheng Biotech Co., Nanjing, China) according to the manufacturer’s instructions. Four fish larvae from each treatment were homogenized in a cold buffer, centrifuged, and the supernatant was diluted according to the kit instructions. The corresponding reagents were then introduced, and the determinations were conducted using a spectrophotometer.

### 2.7. Behavioral Experiment

The behavioral experiments were conducted according to established protocols [34,35]. On the 7th and 14th day of exposure, 10 fish larvae were randomly selected from each treatment group for behavioral analysis. Prior to filming, the larvae were acclimated for 30 min to minimize stress. A suitable filming orientation, free from reflections and optical distortions, was identified, and a cell phone was securely fixed with a bracket to ensure stable recording conditions. For each trial, a sterile Petri dish with a diameter of 9 cm was utilized, filled with 1/2 of its volume of the treatment group solution. A single fish from the respective treatment group was randomly introduced into the dish after a 5 min acclimatization period. Each fish was recorded for 5 min, and to prevent cross-contamination, Petri dishes and exposure solutions were replaced for each fish across different treatment groups. A blank control video lasting 10 min was also recorded, featuring only artificial seawater for static shooting. The Smart 3.0 video tracking system and Panlab software were used to analyze the behavioral parameters of each fish, including velocity (cm/s), distance moved (cm), acceleration state center-point (s), and in-zone arena (s).

### 2.8. Statistical Analysis

SPSS 26 was used for statistical analysis, and the significance of differences between the control group and the experimental group was tested by means of one-way ANOVA. Data were expressed as mean ± standard deviation. Differences between all treatments were determined by the least significant difference (LSD) test. *p* < 0.05 was considered to be a significant difference.

## 3. Results

### 3.1. Intake and Excretion of PLA-MPs by Marine Medaka Larvae

**Figure 2:**
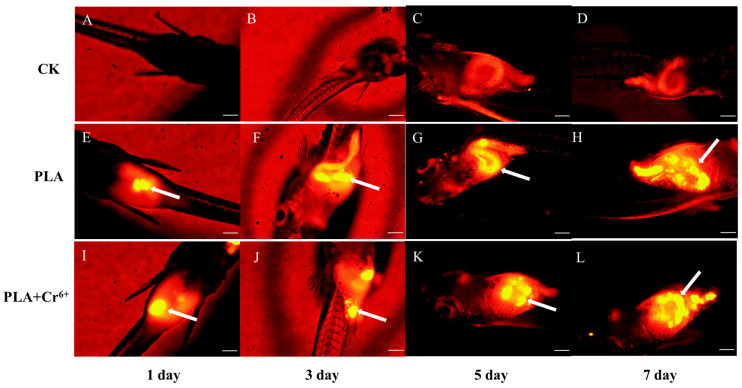
Intake of fluorescent PLA-MPs by marine medaka larvae. The yellow bright spots, as highlighted by white arrows, indicate the presence of fluorescent PLA-MPs (*n* = 3). (**A**–**D**) Intestinal fluorescence microscope photos of control group larvae on days 1, 3, 5, and 7 of the intake experiment; (**E**–**H**) intestinal fluorescence microscope photos of PLA-exposed group larvae on days 1, 3, 5, 7 of the intake experiment; (**I**–**L**) intestinal fluorescence microscope photos of PLA + Cr^6+^-exposed group larvae on days 1, 3, 5, 7 of the intake experiment. The scale is 200 μm.

**Figure 3:**
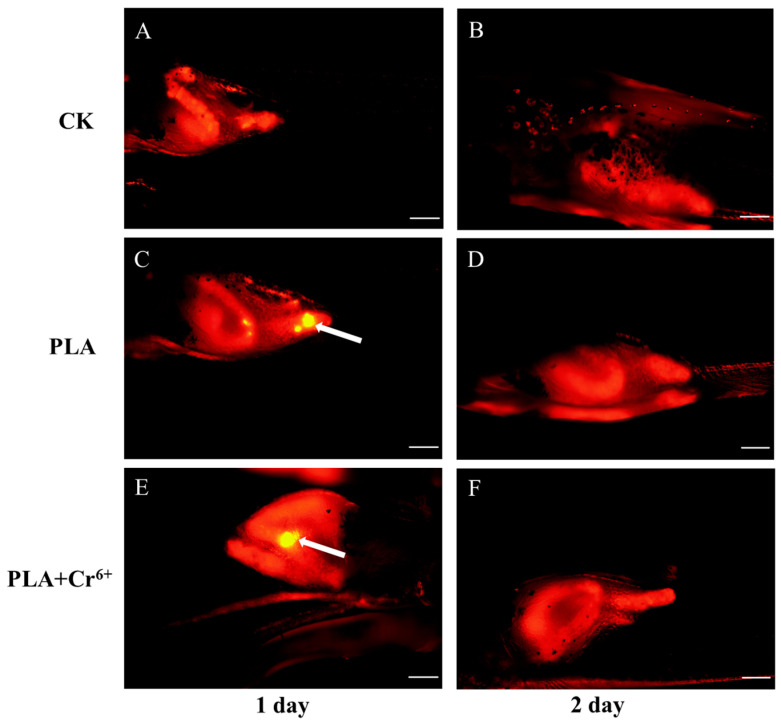
Excretion of fluorescent PLA-MPs by marine medaka larvae. The yellow bright spots, as highlighted by white arrows, indicate the presence of fluorescent PLA-MPs (*n* = 3). (**A**,**B**) Intestinal fluorescence microscope photos of control group larvae on days 1 and 2 of the excretion experiment; (**C**,**D**) intestinal fluorescence microscope photos of PLA-exposed group larvae on days 1 and 2 of the excretion experiment; (**E**,**F**) intestinal fluorescence microscope photos of PLA + Cr^6+^-exposed group larvae on days 1 and 2 of the excretion experiment. The scale is 200 μm.

Figure 2 shows the intake and excretion of PLA-MPs by marine medaka larvae. Compared with the control group, fluorescent PLA-MPs were detected in the larvae of the two treatment groups on the 1st day of exposure, with a significant increase in fluorescent PLA-MPs’ enrichment over time (Figure 2E–L). Notably, the results show that PLA-MPs can be excreted rapidly. A comparison of images taken on the 7th day of the intake experiment (Figure 2H,L) with those taken on the 1st day of the excretion experiment (Figure 3C,E) reveals a substantial decrease in fluorescent PLA-MPs within the larvae. Furthermore, by the 2nd day of the excretion experiment, no fluorescent PLA-MPs were detected in larvae from any exposed group (Figure 3D,F).

### 3.2. Effects of Individual and Combined Exposure on the Growth of Marine Medaka Larvae

**Figure 4:**
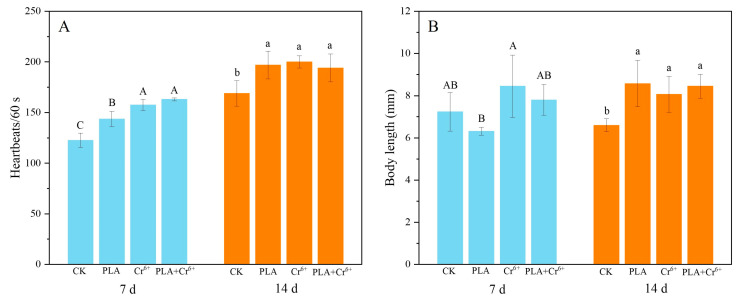
Effects of different treatments on the growth and development of marine medaka larvae. Note: CK: control; PLA: polylactic acid; Cr^6+^: chromium; PLA+ Cr^6+^: polylactic acid + chromium. (**A**) Effects of different treatments on the heartbeat of larvae (*n* = 3); (**B**) effects of different treatments on the body length of larvae. Different letters indicate significant differences among treatments (*n* = 8, *p* < 0.05).

Exposure to PLA-MPs and Cr^6+^ individually and in combination resulted in a significant increase in heart rate in marine medaka larvae (Figure 4A). After 7 days of exposure, no significant differences in body length were observed among the treatment groups. However, after 14 days of exposure, both individual and combined exposures resulted in a significant increase in body length compared to the control group (Figure 4B). The body length of the marine medaka larva increased from 6.24–6.76 mm to 7.32–9.00 mm.

### 3.3. Effects of Individual and Combined Exposure on the Intestinal Tract of Marine Medaka Larvae

**Figure 5:**
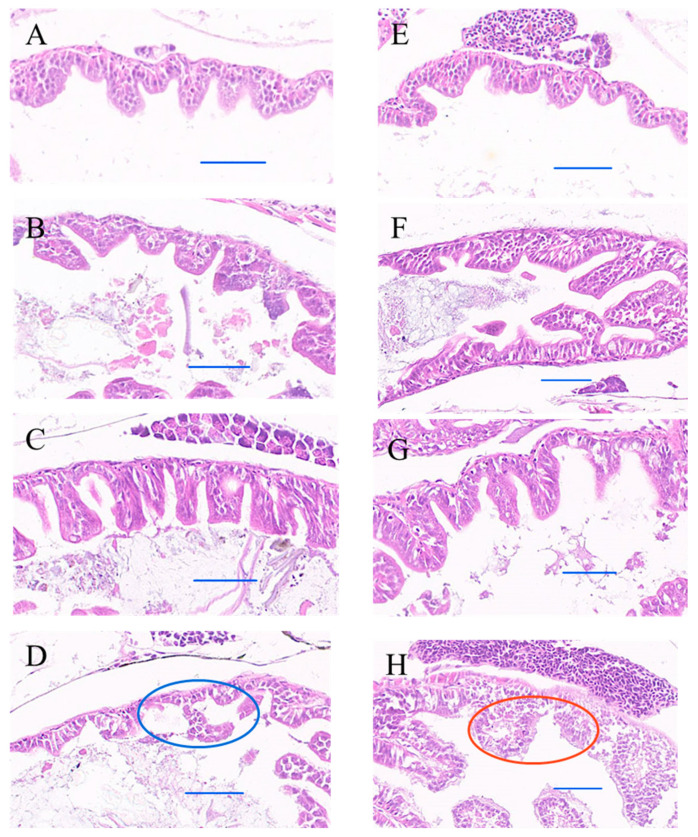
Intestinal tissue damage in marine medaka larvae under different treatments. (**A**) Control group at 7 days; (**B**) PLA-MP exposure at 7 days; (**C**) Cr^6+^ exposure at 7 days; (**D**) combined exposure to PLA-MPs and Cr^6+^ at 7 days; (**E**) control group at 14 days; (**F**) PLA-MP exposure at 14 days; (**G**) Cr^6+^ exposure at 14 days; (**H**) combined exposure to PLA-MPs and Cr^6+^ at 14 days. Note: The red circle denotes villi fusion, the blue circle indicates villi shedding, and the blue line segment indicates a scale of 50 μm (*n* = 3).

**Figure 6:**
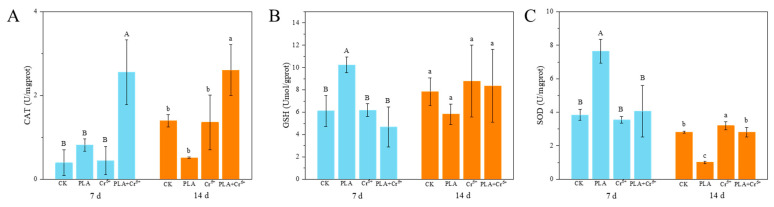
Effects of different treatments of antioxidant enzyme activity in the intestinal tract of marine medaka larva. Note: CK: control; PLA: polylactic acid; Cr^6+^: chromium; PLA+ Cr^6+^: polylactic acid + chromium. (**A**) CAT enzyme activity; (**B**) GSH enzyme activity; (**C**) SOD enzyme activity. Different letters indicate significant differences among treatments (*n* = 4, *p* < 0.05).

Combined exposure to PLA-MPs and Cr^6+^ caused damage to the intestinal tissue of the larvae, characterized by loss of intestinal villi (Figure 5D) and lysis atrophy (Figure 5H). Furthermore, differences in antioxidant enzyme activities were observed in fish larvae. Specifically, CAT activity increased significantly to 2.55 ± 0.774 U/mgprot and 2.60 ± 0.62 U/mgprot on the 7th and 14th days of combined exposure, respectively (Figure 6A). In contrast, no obvious changes were observed in SOD and GSH (Figure 6B,C). Exposure to PLA-MPs led to a noteworthy rise in GSH levels on the 7th day (10.24 ± 0.69 umol/gprot), which returned to normal by the 14th day. SOD activity showed a significant increase on the 7th day (7.65 ± 0.71 U/mgprot), followed by a pronounced decrease on the 14th day (1.02 ± 0.072 U/mgprot) (Figure 6B,C). Conversely, exposure to Cr^6+^ had a minimal impact on enzyme activity, with the exception of a noteworthy increase in SOD activity observed on the 14th day (Figure 6C).

### 3.4. Effects of Individual and Combined Exposure on the Behavior of Marine Medaka Larvae

**Figure 7:**
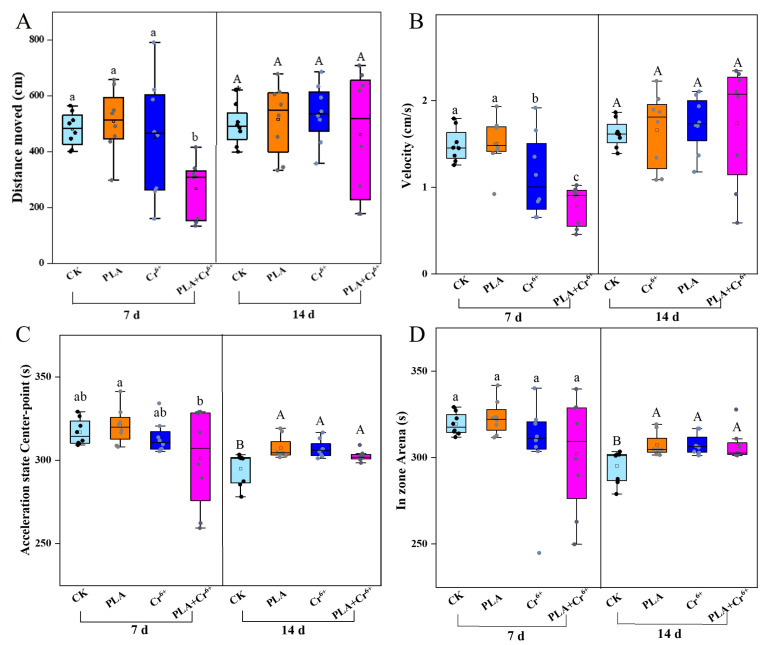
Effects of different treatments on the behavior of marine medaka larvae. Note: CK: control; PLA: polylactic acid; Cr^6+^: chromium; PLA+ Cr^6+^: polylactic acid + chromium. (**A**) Effects of exposure to different treatments on larvae velocity; (**B**) effects of exposure to different treatments on larvae distance moved; (**C**) effects of exposure to different treatments on larvae acceleration state; (**D**) effects of exposure to different treatments on larvae in-zone arena. Different letters indicate significant differences among treatments (*n* = 10, *p* < 0.05).

The locomotive behavior of marine medaka larvae was significantly impacted by combined exposure on the 7th day, with a significant decrease in velocity and distance moved (Figure 7A,B). However, by the 14th day, the swimming behavior had returned to levels consistent with the control group. Two metrics, i.e., the acceleration state center-point and in-zone arena, were used to assess the anxious behavior of the fish larvae. Compared to the control group, both parameters showed a significant increase on the 14th day, indicating that both individual and combined exposure resulted in anxious behavior in the marine medaka larvae (Figure 7C,D).

## 4. Discussion

The aquatic environment is characterized by complex interactions among various pollutants, highlighting the need to study the impacts of combined exposure to MPs and heavy metals on aquatic organisms [26]. Previous studies indicate that MPs can exacerbate the toxicity of Cr^6+^, even at environmental levels [36]. Consistent with these findings, our study revealed that 14-day exposure to low concentrations of PLA-MPs and Cr^6+^, individually and in combination, exerted toxic effects on marine medaka larvae. Notably, combined exposure exhibited stronger toxic effects than single exposure.

### 4.1. Effects on Growth

PLA is considered an ideal alternative to traditional petroleum-based MPs due to its biodegradability under natural conditions. The intake of MPs can cause digestive system blockages, inducing satiety and reducing normal food intake, thus affecting organism growth [37]. Zimmermann et al. showed that exposure to 500 mg/L PLA-MPs inhibited growth, significantly reduced average body length, and caused mortality rates of up to 60% in adult Daphnia magna in aquatic environments [38]. In this study, there was no mortality, and individual and combined exposure did not have a significant negative effect on the growth of marine medaka larvae, except for a notable increase in heart rate (Figure 4A,B). This is due to, on the one hand, different species having different tolerances to PLA [13]. For instance, Duan et al. exposed zebrafish to PLA for 15 days and observed no mortality or weight changes [10]. On the other hand, the discrepancy may be due to differences in MP concentrations. Previous studies have shown that the mass concentration (particle number) of MPs is a key factor affecting toxicity [39]. The concentrations of PLA (100 μg/L) and Cr (50 μg/L) used in this study were close to ambient concentrations [33,40], which may be an important factor contributing to the lack of significant negative effects observed in marine medaka larvae in our investigations.

### 4.2. Intestinal Damage and Antioxidant Enzyme Activity

The odor and degradability of PLA-MPs can lead to bio-erroneous ingestion by marine organisms [41]. Our study corroborates these findings, demonstrating the presence of PLA-MPs in the intestines of all marine medaka larvae on the 1st day, with a significant increase in PLA-MPs abundance over time (Figure 2E–J). Intestinal damage is a major effect of MPs’ ingestion [42]. In this study, combined exposure to PLA-MPs and Cr^6+^ resulted in intestinal tissue damage in marine medaka larvae (Figure 5D,H). The main reason may be the retention of PLA-MPs in the gut during the feeding phase (Figure 2), with combined exposure potentially increasing the bioavailability and accumulation of Cr^6+^, leading to toxic effects. Liao and Yang found that Cr^6+^ loaded on PLA-MPs showed high bioavailability in three stages of digestion (the stomach, small intestine, and large intestine) using an in vitro human digestion model [43].

Individual and combined exposure to MPs and Cr can induce oxidative stress by changing antioxidant enzyme activity in organisms [44]. Our results indicate that exposure to PLA individually affected the activity of three enzymes, among which SOD exhibited the most significant changes, markedly increasing at day 7 and significantly decreasing at day 14 (Figure 6C). In contrast, combined exposure resulted in a significant increase in CAT enzyme activity (Figure 6A). Exposure to Cr^6+^ individually had the least effect, causing a significant increase in SOD only at day 14 (Figure 6C). Oxidative stress is recognized as a key mechanism in MPs’ toxicity [45], and antioxidant enzymes in organisms play a key defensive role against MP-induced oxidative stress [46]. The migration of MP-bound Cr^6+^ in water may lead to Cr^6+^ bioaccumulation [47], potentially resulting in enhanced oxidative stress effects on larvae under combined exposure conditions.

### 4.3. Influence of Locomotive Behavior

This study reported the effects of PLA-MPs on the locomotive behavior of aquatic organisms. Zebrafish exposed to PLA-MPs exhibited anti-predatory defense responses, as observed by Chagas, and Oliveira et al. found through field experiments that exposure to 3 and 9 mg/L PLA-MPs not only reduced the swimming ability of zebrafish larvae but also induced anxiety-like behaviors [34,48]. In our study, combined exposure to PLA-MPs and Cr^6+^ decreased swimming behavior and led to anxiety-like behaviors in marine medaka larvae, while PLA-MPs exposure individually only induced anxiety-like behaviors (Figure 7). MPs can adsorb heavy metal elements from the environment, potentially altering their environmental behavior and toxic effects on organisms. PLA-MPs can accumulate heavy metal ions from the surrounding environment and release them directly in organisms during migration, causing more pronounced toxic effects [44]. Furthermore, the bioavailability of MPs and their interaction with different environmental pollutants may lead to synergistic toxic effects with other pollutants, such as toxic metals [49,50]. The combined exposure in our study resulted in more severe intestinal damages (Figure 5) and greater changes in antioxidant enzyme activity (Figure 6), which may lead to more significant effects on locomotive behavior observed in marine medaka larvae. Zhang et al. revealed that PLA induced cell death, oxidative stress, and inflammatory responses in mice, leading to changes in various physiological phenomena [51]. Overall, this study offers an initial exploration of the ecotoxicological effects of individual and combined exposure to PLA and Cr^6+^ on marine medaka larvae. Further research is needed for a more comprehensive understanding of the mechanisms associated with intestinal tissue damage, altered oxidative stress effects, and changes in locomotive behavior.

## 5. Conclusions

This study investigated the ecotoxicological effects of individual and combined exposure to PLA and Cr^6+^ on marine medaka larvae through histopathology, biochemical, and behavioral analyses. The results showed that combined exposure induced intestinal tissue damage in fish larvae, while both individual and combined exposure changed oxidase activity and locomotive behavior. Notably, the toxic effects of combined exposure appeared to be more pronounced. In summary, the combined exposure to PLA and Cr at ambient concentrations exacerbated adverse effects on marine medaka larvae, highlighting the potential ecological concerns associated with the combined exposure to biodegradable MPs and heavy metals on marine organism larvae. This study provides a preliminary assessment of the toxic effects of PLA and Cr on marine medaka larvae. Further investigations using transcriptomic and metabolomic techniques are necessary to elucidate the underlying mechanisms of these toxic effects, so as to gain a more in-depth understanding of their impact on marine systems.

## Data Availability

Data will be made available upon request.

## Supplementary Materials

The following supporting information can be downloaded at: https://www.mdpi.com/article/10.3390/toxics12080594/s1, Figure S1: flow chart of the experiment.
