# Supplementary material for: Stressful Effects of Individual and Combined Exposure to Low-Concentration Polylactic Acid Microplastics and Chromium on Marine Medaka Larvae (Oryzias melastigma)
Source: Toxics. 2024 Aug 16;12(8):594. doi: 10.3390/toxics12080594 (PMC11359823; doi:10.3390/toxics12080594)
Supplement: Supplementary file 1 [file toxics-12-00594-s001.zip › toxics-3127484-supplementary.pdf]

Supporting Information

1 Paraffin section making

1.1 The larvae fish was fixed with fixed liquid for more than 24 hours. Remove the larvae fish from the fixed liquid and trim the target tissue with a scalpel in the ventilation cupboard, and put the trimmed tissue and the label in the dehydration box.

1.2 Dehydration and wax leaching: put the dehydration box into the dehydrator in order to dehydrate with gradient alcohol. 75% alcohol for 4 hours, 85% alcohol for 2 hours, 90% alcohol for 2 hours, 95% alcohol for 1 hour, anhydrous ethanol I for 30 min, anhydrous ethanol II for 30 min, alcohol benzene for 5~10 min, xylene II for 5~10 min, 65℃ melting paraffin I for 1h, 65 ℃ melting paraffin II for 1h, 65℃ melting paraffin III for 1 hour.

1.3 The wax-soaked tissue is embedded in the embedding machine. First, put the melted wax into the embedding frame, and before the wax solidifies, remove the tissue from the dewatering box and put it into the embedding frame according to the requirements of the embedding surface and affix the corresponding label. Cool at-20℃ freezing table, and after the wax is solidified, the wax block is removed from the embedded frame and repaired.

1.4 Place the trimmed wax block cool at-20℃ freezing table, slice the modified tissue chip wax block on the paraffin slicer, the slice thickness is 4μm. The tissue is flattened when the slice floats on the 40℃ warm water of the spreading machine, and the tissue is picked up by the glass slides and baked in the oven at 60℃. After the water-baked dried wax is melted, it is taken out and stored at room temperature.

2 HE staining

2.1 Dewaxing as followed: Xylene I for 20 min; Xylene II for 20 min; 100% ethanol I for 5 min; 100% ethanol II for 5 min; 75% ethanol for 5 min; Rinsing with tap water.

2.2 Stain sections with Hematoxylin solution for 3-5 min, rinse with tap water. Then treat the section with Hematoxylin Differentiation solution, rinse with tap water. Treat the section with Hematoxylin Scott Tap Bluing, rinse with tap water.

2.3 85% ethanol for 5 min; 95% ethanol for 5 min; Finally Stain sections with Eosin dye for 5 min.

2.4 Dehydrate as followed: 100% ethanol I for 5 min; 100% ethanol II for 5 min; 100% ethanol III for 5 min; Xylene I for 5 min; Xylene II for 5 min; Finally seal with neutral gum.

2.5 Observe with microscope inspection, image acquisition and analysis.

3 **Flow chart of the experiment**

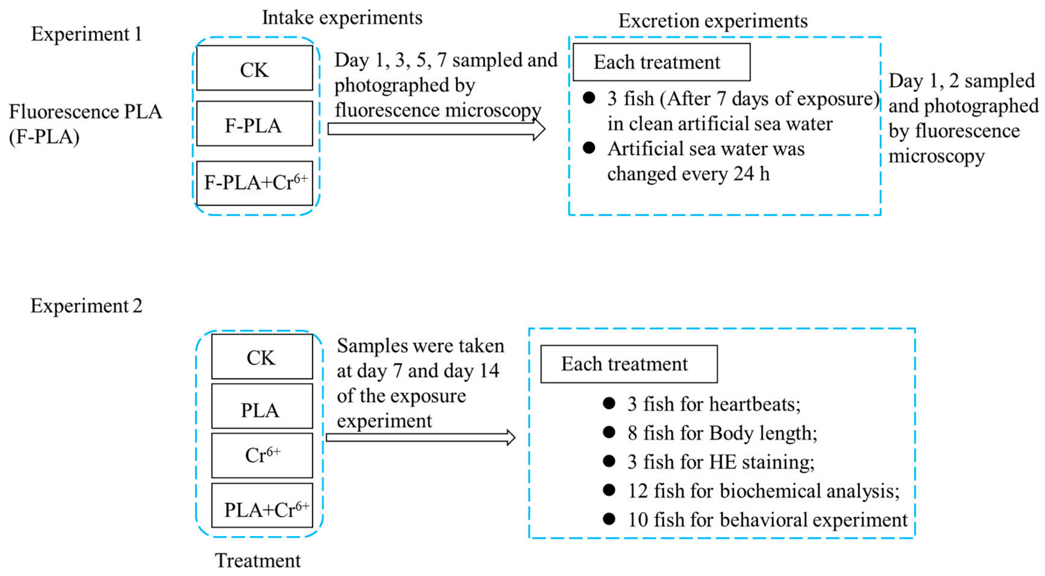

Figure S1. flow chart of the experiment.
